# Strategies in the optimization of DNA hybridization conditions and its role in electrochemical detection of dengue virus (DENV) using response surface methodology (RSM)

**DOI:** 10.1039/d3ra00216k

**Published:** 2023-06-16

**Authors:** Jahwarhar Izuan Abdul Rashid, Nor Azah Yusof, Jaafar Abdullah, Rafidah Hanim Shomiad @ Shueb

**Affiliations:** a Department of Chemistry and Biology, Centre for Defence Foundation Studies, National Defence University of Malaysia Sungai Besi Camp 57000 Kuala Lumpur Malaysia jahwarhar@upnm.edu.my; b Department of Chemistry, Faculty of Science, Universiti Putra Malaysia Serdang Selangor 43400 Malaysia; c Institute for Research in Molecular Medicine (INFORMM), Universiti Sains Malaysia 16150 Kubang Kerian Kelantan Malaysia

## Abstract

In recent years, limited research has been conducted on enhancing DNA hybridization-based biosensor approaches using statistical models. This study explores the application of response surface methodology (RSM) to improve the performance of a DNA hybridization biosensor for dengue virus (DENV) detection. The biosensor is based on silicon nanowires decorated with gold nanoparticles (SiNWs/AuNPs) and utilizes methylene blue as a redox indicator. The DNA hybridization process between the immobilized DNA probe and the target DENV gene was monitored using differential pulse voltammetry (DPV) based on the reduction of methylene blue. Fourier-transform infrared spectroscopy (FTIR) and electrochemical impedance spectroscopy (EIS) were employed to confirm successful DNA hybridization events on the modified screen-printed gold electrode (SPGE) surface. Several parameters, including pH buffer, NaCl concentration, temperature, and hybridization time, were simultaneously optimized, with NaCl concentration having the most significant impact on DNA hybridization events. This study enhances the understanding of the role of each parameter in influencing DNA hybridization detection in electrochemical biosensors. The optimized biosensor demonstrated the ability to detect complementary oligonucleotide and amplified DENV gene concentrations as low as 0.0891 ng µL^−1^ (10 pM) and 2.8 ng µL^−1^, respectively. The developed biosensor shows promise for rapid clinical diagnosis of dengue virus infection.

## Abstract 2

Schematic of the fabrication and mechanism detection of our developed biosensor.

## Graphical abstract

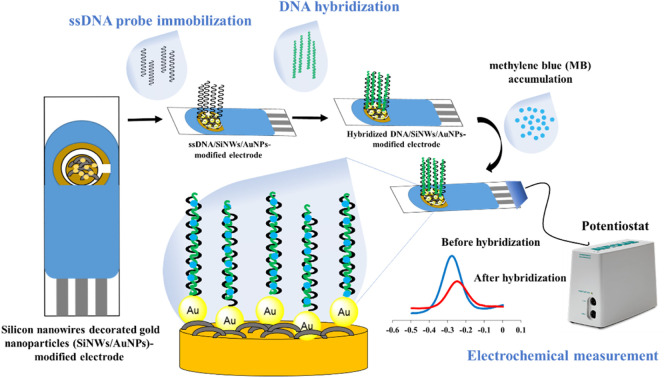

## 1. Introduction

In recent years, electrochemical biosensors have emerged as a promising platform for developing point-of-care (POC) devices for the rapid diagnosis of infectious diseases. These devices offer advantages such as ease of miniaturization, simplicity, versatility, and cost-effectiveness. Moreover, previous research has demonstrated that electrochemical approaches can achieve femtomolar or attomolar concentrations, which is beneficial for the clinical diagnosis of infectious diseases.^1,2^ Coupling POC devices with miniaturized electrochemical transducers has transformed the detection landscape, enabling real-time, rapid detection of various infectious diseases, including COVID-19,^3^ dengue virus (DENV),^4^ Zika virus,^5^ Influenza,^6^ Hepatitis,^7^ human immunodeficiency (HIV),^8^ Salmonella^9^ and Tuberculosis.^10^

Electrochemical biosensors rely on changes in measurable redox current due to specific interactions between immobilized biological recognition elements and analytes, such as ssDNA/RNA-ssDNA, aptamer-antigens/proteins, antibodies-antigens, and whole cells-antigen/proteins. Among these, DNA hybridization, involving the interactions between ssDNA/RNA and its complementary target sequence, is widely used in electrochemical sensing for detecting specific DNA sequences. High accessibility of complementary targets to DNA probe-modified electrode surfaces for DNA hybridization plays a vital role in enhancing electrochemical DNA hybridization detection.

In addition to depending on the improvement of the optimal conditions of the hybridization process, the sensing layer for immobilization and hybridization also needs to be considered.

A good sensing layer can enhance immobilization and DNA hybridization, influencing the biosensor's electrochemical signal.

Numerous studies have improved biosensor performance by using nanomaterials such as multiwalled carbon nanotubes (MWCNTs),^11^ zinc oxide nanoparticles,^11^ gold nanoparticles,^12^ carbon dots Fe_3_O_4_,^13^ silica nanoparticles,^14^ Au–Pt bimetallic nanoparticles/graphene oxide^15^ and *etc.* as a sensing layer in DNA biosensors fabrication.

In the past few years, our group has focused on electrode modification using hybrid nanomaterials consisting of silicon nanowires (SiNWs) and gold nanoparticles (AuNPs). We found that SiNWs/AuNPs nanocomposites could discriminate electrochemical signals with and without the presence of dengue virus gene, making them suitable for use as sensing materials.^3,15–17^ Despite limited research on SiNWs in electrochemical DNA detection, their unique features, such as strong conductivity, high biocompatibility, and high surface-to-volume ratio, make them promising for this application. Although we have successfully enhanced DNA probe immobilization on SiNWs/AuNPs modified electrodes, there is room for improvement in biosensor sensitivity and performance. This includes enhancing the DNA hybridization process on the modified electrode.

Optimizing DNA hybridization conditions is crucial for achieving high sensitivity and selectivity in DNA sensors.^16^ Hence, the optimization of DNA hybridization condition is needed. These parameters include pH, ionic strength, hybridization temperature, and time. Careful control of these parameters can optimize the sensitivity and specificity of the biosensor, enabling accurate detection of target DNA sequences in a sample. Although there have been numerous studies looking into and highlighting the optimum value of the DNA hybridization parameters, most rely on the one-factor-at-a-time method, (changing one parameter at a time while maintaining the other parameters at a constant level), which has drawbacks. This approach cannot evaluate the effects and interactions of parameters on the response, and there is limited understanding of the role these parameters play in response behavior. Thus, one of promising strategies to overcome the above issue is the application of statistical modelling design known as response surface methodology (RSM) in the field of biosensor to optimize their performance.^17,18^ RSM involves using statistical techniques in the design of experiments, development of models, evaluation of key variables, and prediction of responses under optimal conditions. One of the unique aspects of using RSM is that it allows researchers to identify the combination of factors that will optimize the sensitivity, selectivity, and stability of the biosensor. This approach also enables researchers to identify the most important factors, as well as reduce the number of experiments required to identify the optimal combination of factors.^19^ Regarding practical application value, our previous study shows that the developed biosensor, which relies on the DNA hybridization principle, has the potential to act as a diagnostic platform for various infectious diseases. This allows for quicker detection and timely, suitable treatment. Additionally, the RSM optimization method employed in this research can be applied to numerous biosensing applications, thus contributing to the broader field of electrochemical biosensor development and optimization. To our knowledge, there is limited research focusing on optimizing DNA hybridization conditions for DNA electrochemical sensing applications. The relationship between parameters in DNA hybridization conditions and their impact on DNA biosensor performance is still poorly understood. Therefore, this study aims to utilize RSM to explore the behaviour of parameters in DNA hybridization conditions, shedding light on their influence on the efficiency and specificity of DNA hybridization. We applied the RSM approach to optimize DNA hybridization conditions such as pH buffer, NaCl concentration, temperature, and hybridization time, aiming to enhance the electrochemical current signal for detecting dengue virus, which was used as the main analyte in this work.

## 2. Experimental procedure

### 2.1 Reagents and apparatus

Silicon nanowires suspension with average diameter of 150 nm and average length of 20 µm, gold chloroauric acid salt (HAuCl_4_·4H_2_O), potassium ferricyanide (iii) [K_3_Fe(CN)_6_], sodium citrate (Na_3_C_6_H_5_O_7_), (3-aminopropyl) triethoxysilane (APTES), 3-3′-dithiopropionic acid (DTPA), hydrogen peroxide (H_2_O_2_) (30% w/w in H_2_O) and ammonium hydroxide (NH_4_OH) (30% w/w), were purchased from Sigma-Aldrich (USA). Methylene blue (MB) was purchased from R&M Chemicals (Essex, UK). The oligonucleotides sequences of DENV which is based on study of Callahan *et al.*^20^ were purchased from First BASE Laboratories Sdn Bhd, Selangor, Malaysia. For DNA hybridization studies, the TE buffer (0.01 M Tris–HCl; pH 8.0 and 0.001 M EDTA) was used to dilute oligonucleotide stock solutions and remove unbound of dsDNA oligonucleotides. For electrochemical measurement, methylene blue (MB) solution (R&M Chemicals, UK) was prepared as a redox indicator containing 50 M of supporting electrolyte.

The oligonucleotides sequence of dengue virus used in this study; thiolated probe DNA (5′ SH-(CH_2_)_6_-AAC AGC ATA TTG ACG CTG GGA GAG ACC-3); complementary target DNA (5′-GGT CTC TCC CAG CGT CAA TAT GCT GTT-3); one-base mismatch (5′-GGT CTT̲ TCC CAG CGT CAA TAT GCT GTT-3′); three-base mismatch (5′-GGT CTT̲ TCC CT̲G CGT CAA TAT GCA̲ GTT-3′) and non-complementary (5′-TTC TGT GTT AGT ATC TGG GCC ATG TCC-3′). The calculated Δ*G* for the hybridization between the sample DNA and the probe is approximately −37.8 kcal mol^−1^. This negative value indicates that the hybridization process is thermodynamically favorable and spontaneous. Screen printed gold electrode (SPGE) (Dropsens, Spain) based-three electrode system; silver counter electrode and gold electrodes as working and counter electrode, respectively. µAUTOLAB (III) potentiostat (Eco Chemie, Utrecht, The Netherlands) connected to the computer was used to conduct the electrochemical analysis, which was were operated using (GPES) software version 4.9 (Eco Chemie, Netherlands).

### 2.2 Fabrication of the SPGE surface, DNA immobilization, and hybridization

**Fig. 1:**
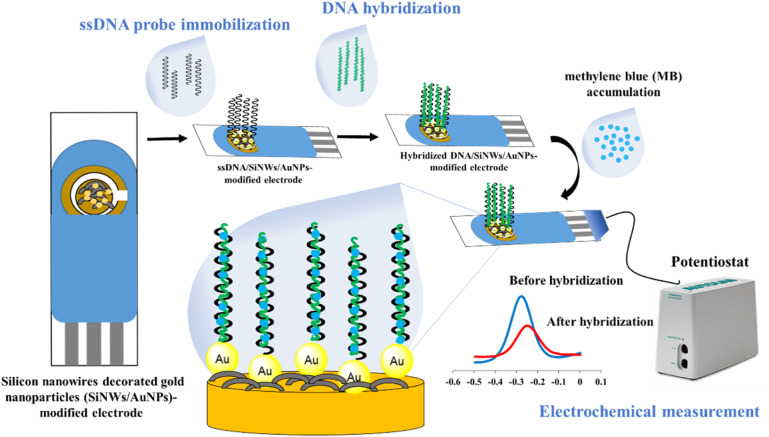
Systematic of the fabrication and mechanism detection of our developed biosensor.

The fabrication of the SPGE surface using SiNWs/AuNPs nanocomposites for our developed DNA biosensor was based on our previous work^21^ as shown in Fig. 1. The working gold electrode surface was drop-casted with 6 µL of SiNWs suspension in 0.5% APTES solution, and dried for 3 hours at room temperature. It was then rinsed with ethyl-ethanol and cured for 30 minutes at 100 °C. The SiNW-modified SPGE was decorated with gold nanoparticles suspension as described in our previous work.^2^ To construct the biorecognition interface for the target dengue virus gene on the fabricated electrode surface, 10 µL of 5 µM thiolated ssDNA oligonucleotide probe was drop-casted onto the surface and left for 10 hours. The excess immobilized thiolated ssDNA probe was gently rinsed three times with TE buffer before hybridization. For the hybridization process, 10 µL of target DNA was directly drop-casted onto the surface of the thiolated ssDNA probe-modified electrode and incubated for 120 min at 40 °C. The hybridized dsDNA-modified electrode was rinsed with TE buffer and dried with N_2_ gas to remove unbound hybridized dsDNA. The same procedure was employed for the hybridization between immobilized ssDNA probe and other different ssDNA sequences and genomic dengue virus gene from real samples used in this study. The preparation of genomic dengue virus genes from real samples has been described in our previous work.^4^

### 2.3 Electrochemical measurement with methylene blue (MB) as a redox indicator

The hybridization was monitored by immersing the hybridized-modified electrode in 50 µM MB containing 50 mM Tris–HCl at pH 7.6 for 20 min, followed by the measurement of cyclic voltammetry (CV) and different pulse voltammetry (DPV) in 50 mM Tris–HCl at pH 7.6 in the potential ranging from −0.5 to 0 V. At the potential ranges of −0.5 to 0 V, step potential 0.005 V, modulation amplitude of 0.5 V with the interval time of 0.64 s at room temperature. The calculation of hybridization efficiency (%) is calculated based on our previous work
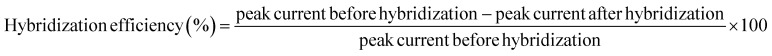


### 2.4 Optimization of DNA hybridization conditions using central composite design (CCD)

Four independent variables, including pH hybridization buffer (6–8), NaCl concentration (1–1.5 M), hybridization temperature (40–45 °C), and incubation time (10–40 min), were applied to the RSM method to determine the optimum condition of the DNA hybridization process on the surface of the fabricated electrode. A total of 30 experiments were suggested according to the central composite design (CCD) using the statistical package, Design-Expert software version 6.0 (Stat-Ease Inc., Minneapolis, USA), with different combinations of parameter values, and hybridization efficiency was taken as the RSM response.

All the experimental data of hybridization efficiency were fitted to the second order polynomial equation below:

1 *Y* = *X*_0_ + *X*_1_*A* + *X*_2_*B* + *X*_3_*C* + *X*_4_*D* + *X*_5_*A*^2^ + *X*_6_*B*^2^ + *X*_7_*C*^2^ + *X*_8_*D*^2^ + *X*_9_*AB* + *X*_10_*AC* + *X*_11_*AD* + *X*_11_*BC* + *X*_12_*BD* + *X*_13_*CD*

where *Y* is the hybridization efficiency, and *A*, *B*, *C*, *D* represent the pH hybridization buffer, salt concentration, hybridization temperature, and incubation time, respectively. The difference in MB peak current before and after hybridization with the complementary target has been used for the measurement of hybridization efficiency.^22^

## 3. Result and discussion

### 3.1 Characterization of different modified SPGE

#### 3.1.1 FTIR analysis of modified electrodes

**Fig. 2:**
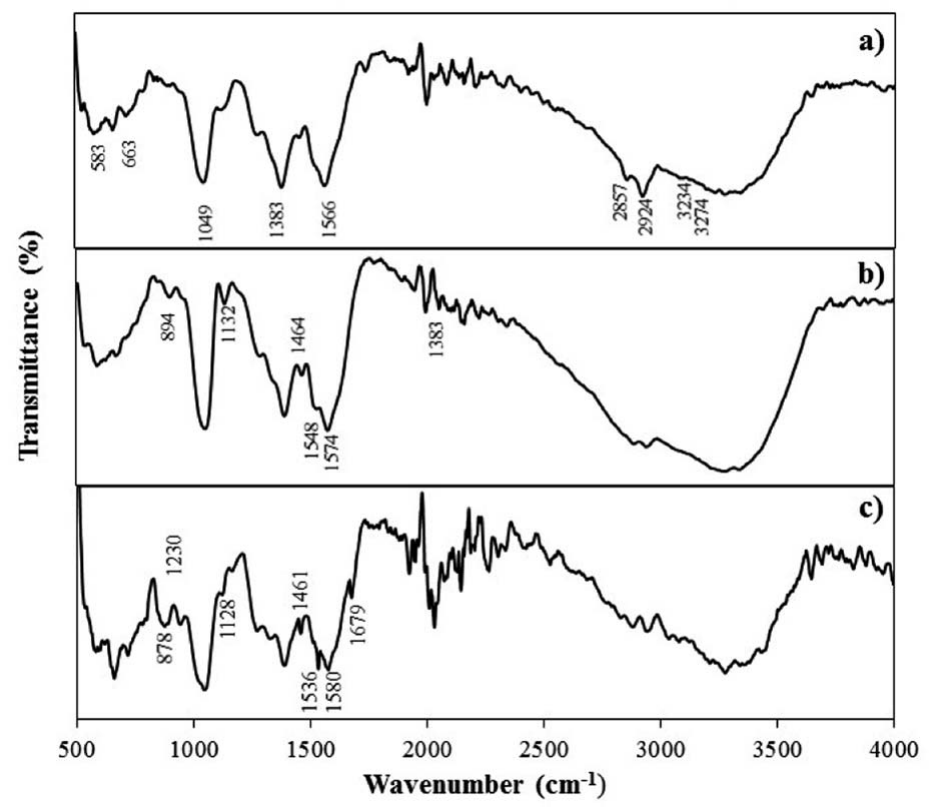
FTIR spectra of (a) modified electrode (b) before hybridization (c) after hybridization.

The Fourier transform infrared (FTIR) was performed to confirm the formation of SiNWs/AuNPs nanocomposites, DNA immobilization and hybridization. Fig. 2 shows that the FTIR spectra of modified electrode exhibits peaks at 1049 cm^−1^, 1383 cm^−1^, 1566 cm^−1^, 2857 cm^−1^, 2924 cm^−1^, 3234 cm^−1^ and 3274 cm^−1^. There are double peaks at 3234 cm^−1^ to 3274 cm^−1^ and 2857 cm^−1^ to 2924 cm^−1^ which corresponded to the stretching mode of NH_2_ and CH_2,_ respectively.^23,24^ The absorption band at 1049 cm^−1^ corresponds to Si-0-Si stretching mode indicating the formation bonds between silane and oxide groups, as well as the polymerization and crosslinking of silane.^25^ In Fig. 2a, the FTIR spectra of the modified electrode display peaks that indicate the successful immobilization of amine (NH_2_)-coated SiNWs on the electrode surface using the silanization process. This confirms the formation of SiNWs/AuNPs nanocomposites on the electrode surface. Upon immobilization of the ssDNA probe onto the SiNWs/AuNPs-modified electrode surface (Fig. 2b), new peaks emerge at 894 cm^−1^, 1132 cm^−1^, 1464 cm^−1^, 1548 cm^−1^, and 1574 cm^−1^. The peaks at 583 cm^−1^ and 663 cm^−1^ represent the Au–S bond, signifying the successful immobilization of gold nanoparticles on thiolated probe DNA.^26^ The peaks at 834 cm^−1^ and 1132 cm^−1^ correspond to the symmetric and asymmetric phosphate (PO_4_^−^) group and the DNA backbone, respectively.^26,27^ Furthermore, the peaks observed at 1464 cm^−1^, 1548 cm^−1^ and 1574 cm^−1^ are assigned to the four nucleotide of DNA namely cytosine (in plane vibration of cytosine), adenine (C_7_=N vibration of adenine), thymine (C_2_=O) and guanine (C=O stretch of guanine) indicating the successful immobilization of the ssDNA probe on the electrode surface.^28^ Similar FTIR peaks were obtained for the ssDNA/AuNPs/SiNWs-modified electrodes after the introduction of complementary DNA target, confirming the presence of DNA immobilization and hybridization (Fig. 2c).

#### 3.1.2 Electrochemical behavior of [Fe(CN)_6_]^3−*/*4−^at different modified electrodes

**Fig. 3:**
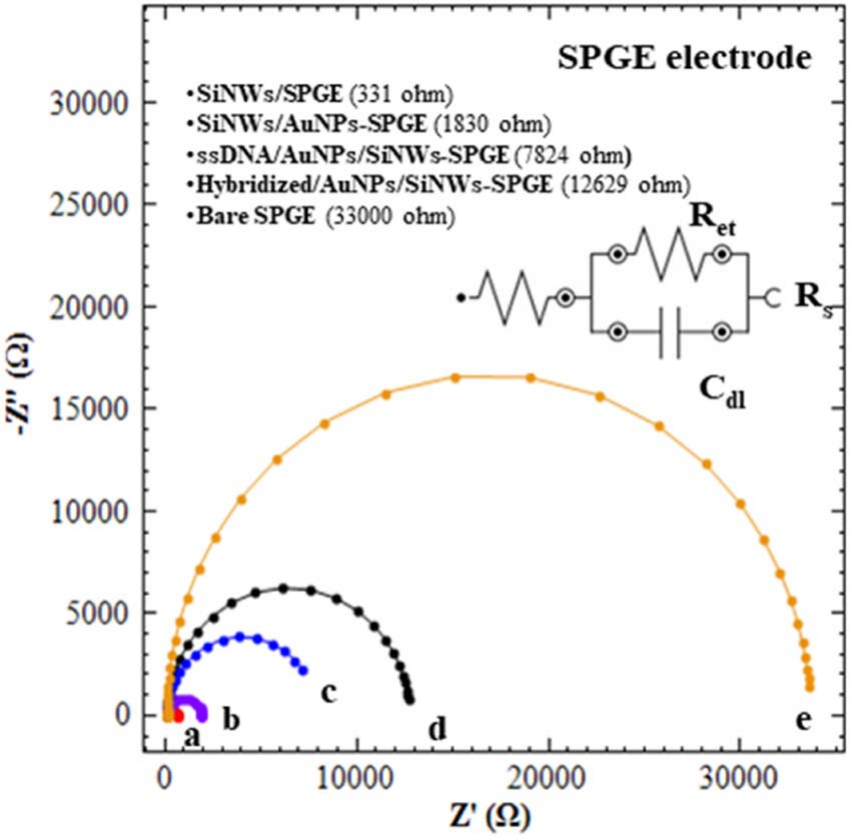
Nyquist plots obtained for different modified SPGE (analysis of (a) bare SPGE, (b) SiNWs/SPGE (c) AuNPs-SiNWs/SPGE, (d) ssDNA probe/AuNPs/SiNWs-SPGE and (e) hybridized/AuNPs-SiNWs/SPGE in 1.0 mM [Fe(CN)6]^3−/4−^ containing 0f 0.1 M KCl at 0.20 V, frequency range 0.1 Hz to 100 KHz at amplitude 5 mV. Inset: equivalent circuit used to fit the EIS data; *R*_s_, solution resistance; *R*_et_, electron transfer resistance and *C*_dll_, double layer capacitance.

The interfacial properties of electron transfer resistance (*R*_et_) on electrode surface during the fabrication process were monitored using the electrochemical impedance spectroscopy (EIS) technique. Fig. 3 shows the Nyquist plots of different modified SPGE electrodes in 1 mM [Fe(CN)_6_]^3−*/*4−^ containing 0.1 M KCl, pH 8 in the frequency range of 0.1 Hz to 100 KHz. A simple equivalent electric circuit was used to fit EIS data for the measurement of electron transfer resistance (*R*_et_) at each modified electrodes, which consisted of solution resistance (*R*_s_) and double layer capacitance (*C*_dll_) (inset Fig. 3). In the Nyquist plot above, the *R*_et_ values of bare SPGE is 33 000 Ω, (curve e). The *R*_et_ value for bare SPGE are dramatically decreased to 555 Ω and 331 Ω after the modification with SiNWs (curve a). This finding confirmed that the SiNWs may play an important role to facilitate electron transfer and thus improving the conductivity of bare electrodes. Curve b demonstrates that the *R*_et_ values of SiNWs/AuNPs-SPGE reach 1830 Ω, which is higher than curve a, primarily due to the modification of the SiNWs surface with APTES solution. This modification facilitates the formation of a self-assembled monolayer (SAM) and subsequent AuNPs decoration. Although the formation of SAM is crucial for AuNPs immobilization, it also influences the electron transfer process. As compared to curve c, the *R*_et_ values of curve d that kept increased suggested that the ssDNA (DNA probe) was successfully immobilized on SiNWs/AuNPs-SPGE. A further increase in *R*_et_ values of 12 629 Ω (hybridized SiNWs/AuNPs-SPGE) was obtained. These increases of *R*_et_ values were due to the introduction of target DNA for hybrid DNA formation (curve d).

### 3.2 The optimization of DNA hybridization parameters using response surface methodology (RSM)

#### 3.2.1 Model fitting and ANOVA analysis

The hybridization parameters were optimized using the central composite design (CCD) combined with RSM. A total of 30 experiments with different combinations of four parameters – pH buffer, NaCl concentration, hybridization temperature, and time – including six center points were carried out according to the central composite face-centered design (Table 1). Experiment 25 (pH 7, 0.75 M NaCl, 42.5 °C, and 12.5 min) exhibited the highest hybridization efficiency.

**Table 1:** Central composite design (CCD) for DNA hybridization optimization and results of experimental data

Run	pH buffer	NaCl concentration (M)	Temperature (°C)	Hybridization time (min)	Hybridization efficiency (%)	
					Experimental value	Predicted value
1	7	0.5	42.5	12.5	52.67	52.92
2	6	0.5	40	20	43.03	45.35
3	8	0.75	42.5	12.5	58.04	59.14
4	8	0.5	45	5	43.44	42.18
5	6	1	45	20	45.12	43.73
6	6	0.75	42.5	12.5	55.67	56.47
7	6	0.5	45	20	47.05	44.45
8	8	1	40	5	43.34	44.34
9	6	0.5	45	5	39.04	40.51
10	6	1	40	20	43.67	43.62
11	6	1	40	5	39.65	37.68
12	8	1	45	5	52.67	51.45
13	6	1	45	5	45.3	46.29
14	7	0.75	42.5	12.5	61.86	60.19
15	8	0.5	40	5	36.34	36.07
16	8	1	40	20	48.45	47.29
17	7	0.75	42.5	12.5	59.78	60.19
18	7	0.75	42.5	12.5	62.34	60.19
19	8	0.5	45	20	40.41	43.12
20	7	0.75	40	12.5	53.34	56.25
21	7	0.75	42.5	20	55.05	55.03
22	8	1	45	20	46.34	45.9
23	8	0.5	40	20	47.9	45.51
24	7	1	42.5	12.5	55.34	56.7
25	7	0.75	42.5	12.5	63.76	60.15
26	7	0.75	45	12.5	60.12	60.15
27	7	0.75	42.5	5	50.34	51.58
28	7	0.75	45	12.5	61.35	59.36
29	7	0.75	42.5	12.5	58.05	60.15
30	6	0.5	40	5	33.65	32.90

Table 2 presents the analysis of variance (ANOVA) of the quadratic model fitted to our experimental data. The model is highly significant, with a low probability value (<0.0001) for the fabricated electrode and a high *F*-value of 23.87, indicating that the quadratic model fits well and is adequate for our experimental data. A non-significant lack of fit *F* value of 1.56 was obtained in this study. The estimated regression coefficient, *R*^2^ of 0.957 and adjusted *R*^2^ of 0.9169 were obtained. The *R*^2^ values are close to one, indicating a good correlation between experimental data and predicted response. In this study, the low coefficient of variation (CV) of 4.92 indicates good reliability for both models. The adequate precision for the developed model in this study was found to be 15.71, which can be used to navigate the design space.^30^

**Table 2:** ANOVA analysis for quadratic equation modelling of studied parameters on DNA hybridization condition

Sources	Sum of squares	df	Mean square	*F*-Value	*P*-Value
Model	60.19	14	144.17	23.87	<0.0001
*A*	1.3	1	32	5.3	0.0361
*B*	1.89	1	64.22	10.63	0.0053
*C*	1.56	1	43.22	7.21	0.017
*D*	1.72	1	53.39	8.84	0.0095
*A* ^2^	−2.39	1	14.75	2.44	0.139
*B* ^2^	−5.39	1	75.16	12.44	0.003
*C* ^2^	−2.39	1	14.75	2.44	0.139
*D* ^2^	−6.89	1	122.85	20.34	0.0004
*AB*	0.88	1	12.85	2.03	0.1749
*AC*	−0.38	1	2.25	0.37	0.5508
*AD*	−0.75	1	9	1.49	0.2411
*BC*	0.25	1	1	0.17	0.6899
*BD*	−1.62	1	42.25	6.99	0.0184
*CD*	−2.12	1	72.25	11.96	0.0035
Residual	—	15	6.04		
Lack of fit	—	10	6.86	1.56	0.3258
Pure error	—	5	4.4		
Cor total	—	29			
*R* ^2^ = 0.957	CV = 4.92			Adeq precision = 15.705	
*R* ^2^ adjusted = 0.9169					
PRESS = 490.05					

In Table 2, all the linear coefficient terms (*A*: pH buffer, *B*: NaCl concentration, *C*: hybridization temperature, *D*: hybridization time), quadratic coefficient terms (*A*^2^, *B*^2^), and interaction coefficient terms (*BC*, *BD*, *CD*) were statistically significant (*P* < 0.05). From this ANOVA analysis, there is a mutual interaction between NaCl concentration and hybridization temperature, NaCl concentration, and hybridization time, as well as hybridization temperature and time.

All of the experimental data for fabricated electrodes was fitted into the second-order full polynomial equation by applying multiple regression analysis as follows:

2 Hybridiation efficiency (%) = 60.19 + 1.33*A* + 1.89*B* + 1.56*C* − 1.72*D* − 2.39*A*^2^ − 5.39*B*^2^ − 2.39*C*^2^ − 6.89*D*^2^ − 0.38*AC* − 0.75*AD* + 0.25*BC* − 1.62*BD* − 2.12*CD*

where *A* represents pH buffer, *B* represents NaCl concentration, *C* represents hybridization temperature, and *D* represents hybridization time. Based on the linear magnitude coefficient, the factor with the most influence on the DNA hybridization process is as follows: NaCl concentration > hybridization time > hybridization temperature > pH buffer. It was shown that NaCl concentration was the most critical factor for enhancing DNA hybridization events on the fabricated electrode surface.

### 3.3 The effect of hybridization parameters

#### 3.3.1 Effect of pH buffer on hybridization efficiency

**Fig. 4:**
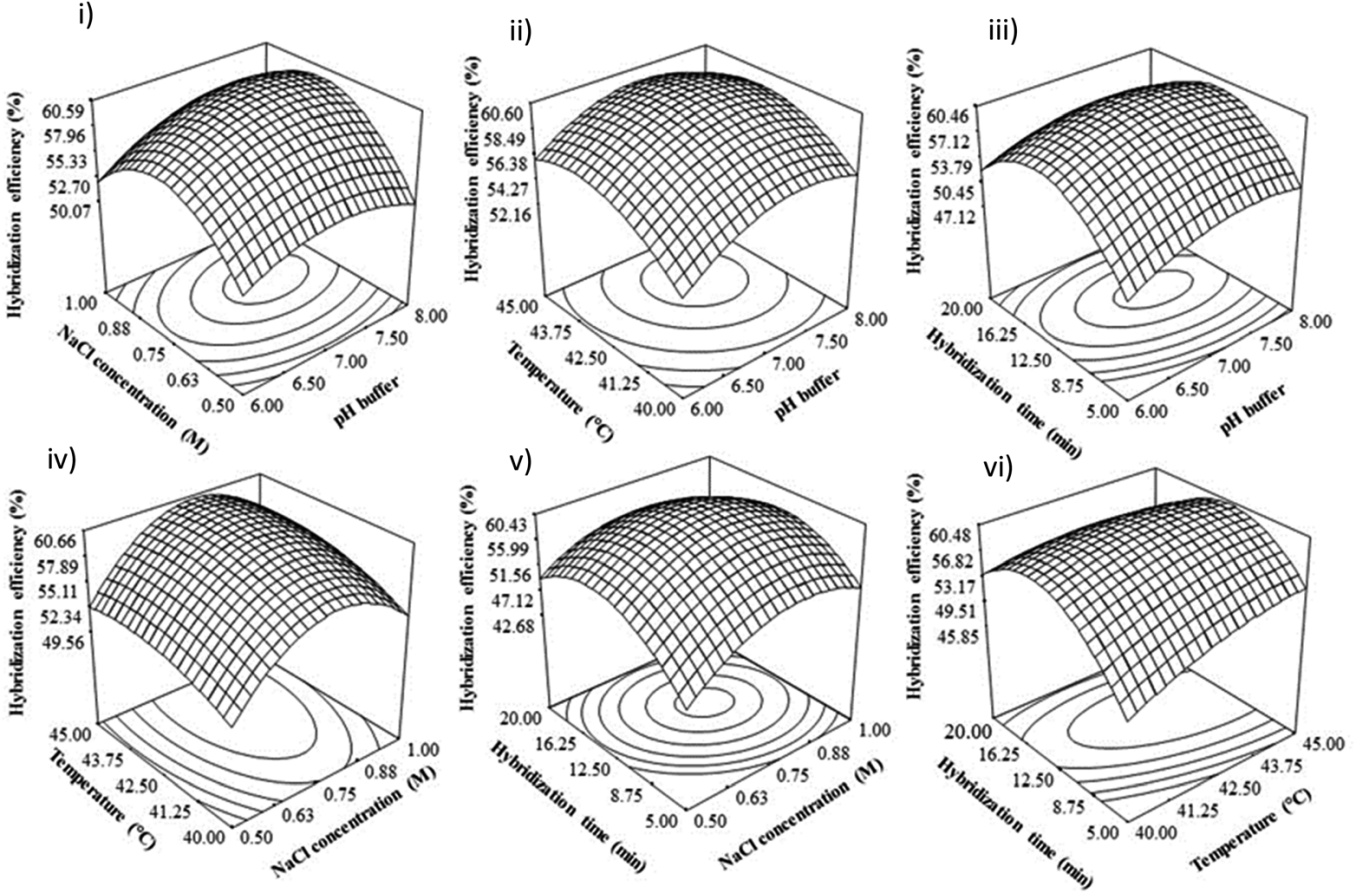
D response graph showing the effect of pH buffer, NaCl concentration, hybridization time and hybridization temperature on hybridization efficiency signal by our developed biosensor. Response surface curve of the influence of the interaction of various factors on the hybridization efficiency; (i) displays the influence of NaCl concentration and pH buffer on the hybridization efficiency; (ii) displays the influence of temperature and pH buffer on the hybridization efficiency; (iii) displays the influence of hybridization time and hybridization temperature on the hybridization efficiency; (iv) displays the influence temperature and NaCl concentration on the hybridization efficiency; (v) displays the influence temperature and NaCl concentration on the hybridization efficiency; (vi) displays the influence hybridization time and NaCl concentration on the hybridization efficiency; (vii) displays the influence hybridization time and temperature on the hybridization efficiency.

The DNA hybridization rate is strongly influenced by the pH of the solution.^29–31^ As shown in the 3D response surface graphs, the DNA hybridization efficiency is enhanced with the increase of pH from 6 to 7.5 and slightly decreases under alkaline conditions (pH 8) as shown in Fig. 4i–iii. In general, all the 3D response surface graphs show that the DNA hybridization efficiency is poor at lower pH (pH 6) and higher pH (pH 8) (Fig. 4i–iii). It is known that the separation of DNA probe strands is governed by the electrostatic repulsion of negatively charged phosphate groups. Hence, an excess of H^+^ in low pH reduces the electrostatic repulsion between DNA probe strands, leading to tensile surface stress where DNA probe strands have a higher tendency to be close together. As a result, hybridization is poor due to the low accessibility of target DNA towards the DNA probe.^32–34^

In alkaline conditions, the hydrogen bond between DNA strands is disrupted, causing the double helix DNA to denature, separating from each other, and forming a single-stranded DNA coil.^35–37^ This can be attributed to the excess of OH^−^ ions in alkaline solutions, resulting in the deprotonation of guanine and thymine bases and breaking the bonding of double helix DNA, which can influence the DNA hybridization rate.^38,39^ Besides that, Wang^47^ found that the electrode sensor would likely be easily damaged under highly acidic or alkaline solutions. Therefore, in this study, the DNA hybridization reaction was suppressed at extreme pH (low pH and high pH), and neutral pH is suitable for duplex DNA formation.

#### 3.3.2 Effect of NaCl concentration on DNA hybridization efficiency

Concentration or ionic strength of NaCl has been observed as the most influential factor on the enhancement of DNA hybridization efficiency as mentioned in the previous ANOVA analysis (Table 2). 3D surface graphs show the effect of NaCl concentration with different combination parameters on the DNA hybridization efficiency of both fabricated electrodes (Fig. 4i, iv and v). In general, the hybridization efficiency signal is enhanced with increasing NaCl concentration from 0.5 M to 0.9 M. This is consistent with previous studies where an increase in salt concentration (cations) was able to stabilize the configuration of hybridized DNA complementary and leading to more DNA hybridization events happening on the electrode surface and solution.^40–43^ This is likely due to the fact that the electrostatic repulsion between the negatively charged phosphate group in the DNA probe and its target is reduced at higher salt concentrations, resulting in a higher hybridization rate.^44^ Whereas, at low ionic strength, the electrostatic repulsion between DNA probe and its target leads to a decrease in hybridization rate. At low ionic strength, the electrostatic repulsion between the DNA probe and its target leads to a decrease in hybridization rate. Furthermore, the ionic strength solution also depends on the interaction of hybridization redox indicator with DNA double helix.^45^ Previously, it has been reported that the redox indicators demonstrated an electrostatic interaction with hybridized DNA such as Ru(NH_3_)_6_^3+^,^46,47^ FcPF6 and Co(bpy)_3_^3+^, Nile blue,^46^ [Os(bipyridine)_2_Cl]-*co*- and ethylamine redox polymer.^48^ Hence, the binding of the redox indicator towards hybridized DNA would probably be partially replaced by the cations (salts) at a very high ionic strength solution, which could affect the electrochemical signal. It is shown by our 3D response surface graph when the NaCl concentration was adjusted above 0.9 M, resulting in a significant decrease in DNA hybridization efficiency (Fig. 4i, iv and v). This could be due to the reduced amount of MB electrostatically adsorbed on hybridized DNA due to the replacement by the excess of Na^+^ ions. Interestingly, this finding suggests that the electrostatic interaction and guanine base interaction are involved in MB binding to the hybridized DNA. Based on the previous ANOVA analysis (Table 2), there is a significant mutual interaction between NaCl concentration and hybridization time on DNA hybridization efficiency. As there was an increase in NaCl concentration, less hybridization time is needed for achieving the optimal DNA hybridization process. These mutual interactions are explained through the minimization of DNA electrostatic effects, which have been minimized at high ionic strength, leading to the fast formation of hybridized DNA.^40,49^

#### 3.3.3 Effect of temperature on DNA hybridization efficiency

The effects of hybridization temperature on DNA hybridization efficiency for developed DNA sensors are shown in the 3D surface graph (Fig. 4ii, iv and vi). In general, the DNA hybridization reaction on our fabricated electrode surface was greatly improved with the increasing hybridization temperature from 40 °C to 45 °C. This suggests that an elevated hybridization temperature is needed to unfold the DNA probes and strands, making them more accessible to bind with each other, and thus, increasing the hybridization efficiency.^50^ For example, Flechsig and Reske^51^ found out that the DNA hybridization signal was enhanced up to 5 folds at the hybridization temperature of 50 °C compared to 23 °C. Our findings revealed that the biosensor we developed exhibited an optimal hybridization temperature between 40 °C and 45 °C for DNA hybridization signals, which is consistent with numerous previous studies.^32,52–55^ In contrast, optimal levels of DNA hybridization events for DNA electrochemical detection could be achieved below 40 °C.^56–58^ Some earlier studies demonstrated that a hybridization temperature above 60 °C is the optimal condition.^59–61^ According to the 3D response surface graph, as the hybridization temperature increases, there is an optimal point at which the hybridization efficiency signal for developed DNA sensors is maximized (Fig. 4vi). This can be attributed to the increased movement of DNA and acceleration of the hybridization kinetic rate at higher temperatures, which in turn, enhances DNA solubility and hybridization efficiency.^33,62,63^ However, higher hybridization temperatures can cause hybridized DNA to become unstable and more prone to dissociation, leading to a decrease in DNA hybridization efficiency.^56,64,65^

#### 3.3.4 Effect of hybridization time on DNA hybridization efficiency

The effects of hybridization time, ranging from 5 to 25 minutes, on DNA hybridization efficiency are shown in 3D response surface graphs (Fig. 4iii, v and vi). Generally, an optimal hybridization time is required, allowing sufficient time for the DNA target to specifically interact with the DNA probe and form hybridized DNA. It was observed that the DNA hybridization signal increased as the hybridization time increased from 5 to 16 minutes, after which it began to decrease (Fig. 4iii, v and vi). This contradicts previous reports, stating that DNA hybridization efficiency increased with hybridization time until reaching a constant state.^66–70^ The constant hybridization signal indicated that the DNA probe on the fabricated electrode surface was fully hybridized with the DNA target, and no further hybridization occurred even with longer hybridization times. However, some previous studies on DNA electrochemical sensors based on methylene blue (MB) reduction signals showed a similar observation to our study, where longer hybridization times reduced the hybridization efficiency signal.^71–76^ It could be assumed that the MB signal might be influenced by the hybridization time.^71,73^ The decrease in hybridization efficiency at longer hybridization times was possibly due to an excess and accumulation of non-binding DNA targets on the electrode surface, which could not be removed by washing steps. Consequently, more guanine bases from non-binding DNA targets were exposed to MB, leading to a high current signal, which reduced the measurement of DNA hybridization efficiency.

### 3.4 Optimization and verification of developed model

An optimal condition for DNA dengue virus detection signals to obtain the maximum DNA hybridization efficiency was determined using Derringer's desired function method in Design Expert 6.06 software. The optimal values of the studied parameters were suggested as pH 7.8, 1.45 M of NaCl concentration, 45 °C, and 10 minutes of hybridization time. Triplicate experiments (*n* = 3) were conducted to validate these optimal conditions, and a DNA hybridization efficiency of 62% (RSD 3.71%) was obtained. These experimental values were in agreement with the predicted values under the suggested optimal conditions. Thus, this optimized condition for DNA hybridization was used for real sample detection.

### 3.5 The selectivity and sensitivity studies of our developed biosensor

**Fig. 5:**
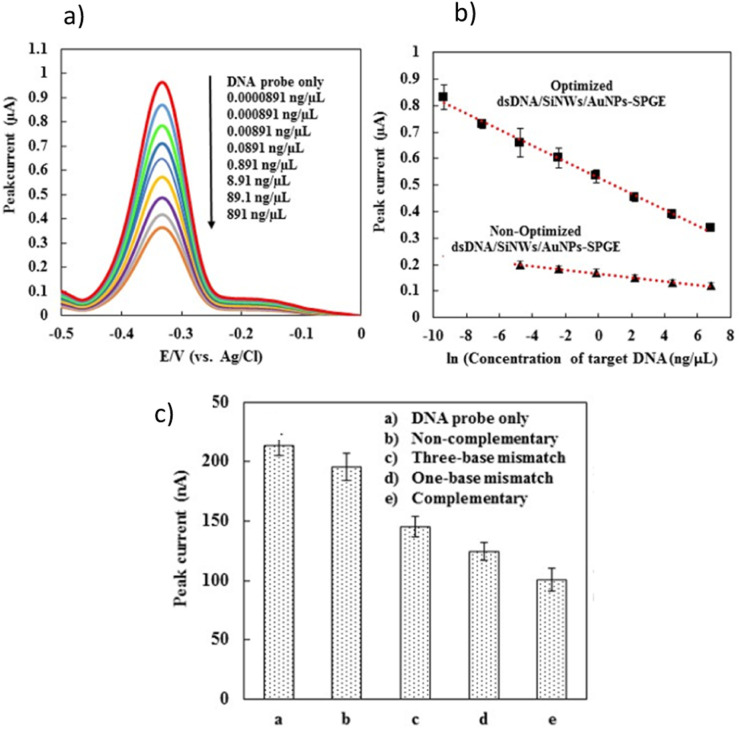
The sensitivity and selectivity studies of developed DNA sensor; (a) DPV response of SiNWs/AuNPs-SPGE at different concentration of target DNA; (b) the comparison of calibration curves for the level of detection before and after optimization by the developed DNA sensors; (c) DPV response of the DNA biosensor for selectivity studies involving non-complementary, single-base mismatch, three-base mismatch, and complementary DNA sequences.

Under optimized condition, the sensitivity of the developed DNA sensor was evaluated by the hybridization of probe DNA with different concentrations of complementary DNA target (Fig. 5a and b). Generally, the MB peak current of developed DNA sensor were decreased linearly with increasing in concentration of the synthetic complementary target. Fig. 5b, demonstrate the linear relationship where the MB peak current are decreased with increasing in concentration of the synthetic complementary DNA target following the equations below, respectively:MB peak current (µA) = −0.0302 × ln(DNA target concentration (ng µL^−1^)) + 0

A cut-off value of 0.89 µA was calculated based on the triplicate analysis (*n* = 3) of the hybridization process between the DNA probe and non-complementary sequences. Consequently, the MB peak current values below the cut-off were interpreted as positive, while those above it were considered negative. Therefore, the lowest concentration of complementary DNA target that yielded an MB peak current below the cut-off value determined the limit of detection (LOD) for our developed DNA sensor. Fig. 5a and c show that the developed DNA sensors were able to detect complementary oligonucleotide dengue virus concentrations as low as 0.0891 ng µL^−1^ (10 pM), respectively. Fig. 5b displays the comparison of calibration curves for the level of detection before and after optimization by the developed DNA sensors. The limit of detection for the developed DNA sensor was significantly improved from 0.00891 ng µL^−1^ (non-optimized) to 0.0000891 ng µL^−1^ under optimized conditions (Fig. 5b). Our developed DNA sensor exhibited a detection limit that can reach the clinical level of RNA dengue virus concentration in blood, which is approximately 10 pM to 10 fM.^77,78^ The selectivity studies for our developed DNA sensor were investigated by hybridizing our developed biosensor with different kinds of synthetic DNA target sequences.

At a concentration of 89.1 ng µL^−1^ (10 nM), various synthetic DNA sequences, such as non-complementary, single-base mismatch, three-base mismatch, and complementary DNA sequences, were tested with our developed biosensor (Fig. 5c). As shown in Fig. 5c, the MB peak current for the non-complementary sequence detection is nearly equal to the MB current obtained for the background signal using a DNA probe without target DNA sequences, indicating that no DNA hybridization occurred. A decrease in MB peak current was observed upon the hybridization of complementary DNA targets (curve e), single-base mismatch (curve d), and three-base mismatch sequences (curve c) due to the inaccessibility of MB binding to the DNA surface, as previously described. The developed DNA biosensor demonstrated a relatively clear signal difference between single-base mismatch and complementary target DNA. This indicates that our DNA biosensor is capable of discriminating between single-base mismatch and complementary target DNA in dengue detection. Generally, the MB peak current generated by developed DNA sensor after hybridization increased in the following order: DNA probe without target > non-complementary DNA > three bases-mismatch > one base-mismatch > complementary sequences. This finding concludes that our newly developed DNA sensor demonstrates good selectivity, as it is able to discriminate between complementary, three base-mismatch, and non-complementary sequence detection.

### 3.6 The analytical performance of developed sensor on real sample detection

**Fig. 6:**
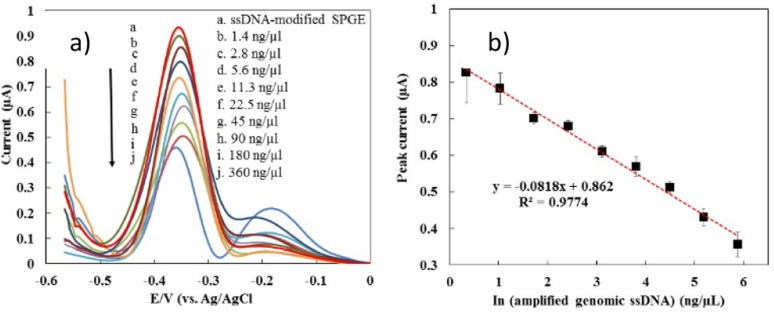
(a) DPV response of SiNWs/AuNPs-modified electrode at different concentration of amplified ssDNA from blood spiked dengue virus; (b) calibration curve of the biosensor response at the different concentration ranging from 1.4 ng µL^−1^ to 360 ng µL^−1^ of genomic ssDNA concentration.

The optimal conditions obtained from the optimization process using the RSM approach were applied to detect actual samples from dengue virus samples. Fig. 6 demonstrates the DPV response of the developed biosensor after hybridization with the genomic ssDNA dengue virus at various concentrations, ranging from 1.4 ng µL^−1^ to 360 ng µL^−1^. It was shown that the MB current decreased with the increase in genomic ssDNA concentration. The detection limit of the developed sensor was estimated based on a cut-off value of 0.89 µA, where the peak current below the cut-off value is interpreted as positive and negative for those above it. The detection limit of our developed sensor was estimated as 2.8 ng µL^−1^. Fig. 6b exhibits that our developed biosensor displayed a linear relationship between the MB peak current and the natural logarithm of the genomic ssDNA concentration (ng µL^−1^), as stated in the equation below:

3 MB current (µA) = −0.08 × ln(genomic ssDNA concentration (ng µL^−1^)) + 0.86

In the previous work, the detection limit of pure synthetic oligonucleotide was 0.000891 ng µL^−1^, which was lower than the obtained detection limit of genomic DNA. As described before, the condition of real samples is very different from the pure synthetic oligonucleotide due to the presence of many impurities of biological molecules that can hinder the hybridization efficiency. In addition, the length of genomic ssDNA is longer and more bulky than the pure synthetic oligonucleotide which could affect the MB binding affinities to the DNA surface and MB current signal.^79^ The reproducibility of the fabricated electrode on detection of genomic ssDNA (5 ng µL^−1^) from serum spiked dengue virus were investigated showed a good reproducibility for nine measurements, where a RSD value of 9.34% and 8.23% were obtained respectively.

## 4. Conclusion

We employed a statistical modelling-based approach called RSM to enhance the sensitivity of our developed biosensor. This approach allowed us to better understand the role and behaviour of different parameters and their impact on the DNA hybridization process. Under optimized conditions, our developed biosensors were able to detect real genomic dengue sequences with a detection limit of 2.8 ng µL^−1^. Our approach, which combines sample preparation with electrochemical detection, is more specific, sensitive, and rapid than traditional methods such as gel electrophoresis visualization and ELISA assays, and is easier to use in practical settings like hospitals and laboratories.

## Conflicts of interest

No conflict of interest.

## Supplementary Material
